# Cost-Consequences Analysis of Increased Utilization of Triple-Chamber-Bag Parenteral Nutrition in Preterm Neonates in Seven European Countries

**DOI:** 10.3390/nu12092531

**Published:** 2020-08-20

**Authors:** Alexander Kriz, Antony Wright, Mattias Paulsson, Stephen Tomlin, Venetia Simchowitz, Thibault Senterre, Julian Shepelev

**Affiliations:** 1 Executive Insight AG, 8008 Zurich, Switzerland; 2 Maverex Ltd., Newcastle upon Tyne NE6 2HL, UK; antonywright@maverex.com; 3 Department of Women’s and Children’s Health, Uppsala University, 751 85 Uppsala, Sweden; mattias.i.paulsson@gmail.com; 4 Great Ormond Street Hospital, London WC1N 3JH, UK; Stephen.Tomlin@gosh.nhs.uk (S.T.); Venetia.Simchowitz@gosh.nhs.uk (V.S.); 5 Department of Neonatology, Liège University, 4000 Liège, Belgium; thibault.senterre@skynet.be; 6 Baxter R&D Europe, Boulevard d’Angleterre 2-4, 1420 Braine-L’Alleud, Belgium; 7 Baxter Healthcare Ltd., Compton RG20 7QW, UK; julian_shepelev@baxter.com

**Keywords:** preterm neonates, compounding errors, parenteral nutrition, ready-to-use three-chamber-bags, cost-consequence analysis

## Abstract

The safety of parenteral nutrition (PN) remains a concern in preterm neonates, impacting clinical outcomes and health-care-resource use and costs. This cost-consequence analysis assessed national-level impacts of a 10-percentage point increase in use of industry-prepared three-chamber bags (3CBs) on clinical outcomes, healthcare resources, and hospital budgets across seven European countries. A ten-percentage-point 3CB use-increase model was developed for Belgium, France, Germany, Italy, Portugal, Spain, and the UK. The cost-consequence analysis estimated the impact on compounding error harm and bloodstream infection (BSI) rates, staff time, and annual hospital budget. Of 265,000 (52%) preterm neonates, 133,000 (52%) were estimated to require PN. Baseline compounding methods were estimated as 43% pharmacy manual, 16% pharmacy automated, 22% ward, 9% outsourced, 3% industry provided non-3CBs, and 7% 3CBs. A modeled increased 3CB use would change these values to 39%, 15%, 18%, 9%, 3%, and 17%, respectively. Modeled consequences included −11.6% for harm due to compounding errors and −2.7% for BSIs. Labor time saved would equate to 41 specialized nurses, 29 senior pharmacists, 26 pharmacy assistants, and 22 senior pediatricians working full time. Budget impact would be a €8,960,601 (3.4%) fall from €260,329,814 to €251,369,212. Even a small increase in the use of 3CBs in preterm neonates could substantially improve neonatal clinical outcomes, and provide notable resource and cost savings to hospitals.

## 1. Introduction

Around 15 million preterm births occur worldwide annually [1] and the rate is increasing, including in developed countries [2,3]. Preterm birth is associated with various modifiable factors (e.g., use of assisted reproductive technology, smoking, alcohol consumption) and non-modifiable factors (e.g., spontaneous multiple gestations, abnormalities of reproductive organs, ethnicity, age) [4]. Despite remarkable progress in improving child survival, every year roughly 1 million preterm neonates die [5,6]. Preterm birth is the leading cause of death globally among children aged younger than 5 years and an important cause of long-term ill health, including disability and non-communicable diseases in later life [6]. Additionally, moderate to late preterm birth (32–37 weeks gestation) and low birth weight (≤2500 g) are associated with developmental delays, raised risk of morbidity and death in later life, and limited socio-emotional competence [7,8,9].

Very preterm births (<32 weeks gestational age) account for around 15% of all premature birth and roughly 2% of births overall [10], but the care and associated costs needed can be substantial. The cost of perinatal healthcare mainly depends on the duration of gestation, and the costs increase exponentially with prematurity. In France, a 2017 national study showed that mean hospitalization costs from the start of pregnancy to 1 year after birth increased from €3926 to €12,026 and €63,547 for neonates born at gestational ages 39–42, 32–36, and 22–27 weeks, respectively [11].

In premature neonates, particularly very premature babies with small weight for gestational age, gut immaturity and clinical instability often do not allow rapid full enteral feeding, leading to insufficient nutritional intake and poor growth [12,13,14]. Parenteral nutrition (PN) is an essential therapy for meeting the high nutritional requirements of such neonates, and to reduce growth restriction, especially during the first weeks of life [12,14,15]. Unfortunately, the timing, quality, and quantity of nutritional intake are not consistently achieved. An international survey performed in 2013 covering 74% of all neonatal intensive care units (NICUs) in France, Germany, Italy, and the UK showed that in 61% the starting lipid dose was too low, as was the protein dose in 60% [16]. Additionally, 40% of NICUs followed a non-recommended caloric target and 38% initiated protein too late [16].

PN is a complex therapy for which compounding is time consuming and a potential source of errors leading to harm. PN is classified by the Institute for Safe Medication Practices (ISMP) as a “high-alert” medication (i.e., has a heightened risk of causing significant harm to patients) that requires specific safeguards [17]. The ISMP recommends independent verification of ingredients for intravenous treatments (medications or nutritional components and diluents), confirmation of the volumes before addition to the final container, and the use of programmable infusion pumps [18]. Errors with intravenous treatments can occur at any stage during ordering, transcribing, verification, compounding, and administration. Keers and colleagues [19] found that human errors (e.g., confusing similar drug or patient names, misreading labels, lapses in concentration, and lack of knowledge) were common contributors to medication errors. However, reporting of medication errors, including those for PN, varies widely. A 2013 survey of nearly 900 hospital healthcare professionals, including pharmacists, dieticians, nurses, and physicians, indicated that 44% of participating institutions did not track PN-related medication errors [20].

PN compounding error rates differ by preparation method. Traditionally, manually compounded PN formulations were worst, reaching up to 37% in one report in 1997, followed by 22% for partially automated compounding [21]. While systems and compounding conditions have changed in the past 20 years, these values represent worst case scenarios. Common errors outside the pharmacy include touch contamination, incorrect calculations of ingredients, and bypassing the built-in safety check systems on automated compounding devices [21]. Conversely, the compounding error rate with fully automated compounders, which are used on a large scale by outsourced compounding centers, is estimated to be 1.7%, and for commercially pre-packaged, ready-to-use PN is <1% [21,22,23]. Bloodstream infections (BSIs) can be an important risk when using PN. During a 48-month study in 2008, the observed weighted BSI risk was 0.66% per PN-day [24]. Later studies showed that ready-to-use PN multi-chamber-bags decreased the infection risk by ≥19% by limiting the number of overall handling steps [25,26].

The French Ministry of Social Affairs, the British Association of Perinatal Medicine, the American Society for Parenteral and Enteral Nutrition (ASPEN), and the ESPGHAN/ESPEN/ESPR/CSPEN guidelines (ESPGHAN = European Society of Paediatric Gastroenterology, Hepatology and Nutrition; ESPEN = European Society for Clinical Nutrition and Metabolism; ESPR = European Society of Paediatric Research; CSPEN = Chinese Society of Parenteral and Enteral Nutrition) currently recommend the use of licensed standardized PN solutions as first-line treatment whenever feasible, including in pediatric patients, and very premature neonates, to improve patient safety and to reduce economic and resource burden [27,28,29,30]. They suggest limiting individualized PN solutions to neonates in whom nutritional requirements are outside the range of available standardized PN formulations [30].

Several industry-prepared three-chamber-bag (3CB) containing standardized PN regimens (termed in this paper as ready-to-use 3CBs) have been proposed for use in premature infants and children, and have been used successfully demonstrating ease of use, provision of adequate nutritional intake, and biological homeostasis and improved growth [31,32,33,34,35]. However, very few health-economic data are available, quality is low, and few studies considered factors, such as prescribing errors, as shown by a UK National Institute for Health and Care Excellence review in 2019 [36]. Cost-consequence analyses enable assessment of changes in care on disaggregated outcome variables such as clinical outcomes, error rates, budget impact, and staff time. The disaggregated outcomes increase transparency of the contributions of different factors that are not as well reflected by cost per case averted or cost per life-year data. Moreover, they reflect the complex considerations of decision making in clinical practice for PN. We used multi-country health-economic modeling to estimate the total national impact on clinical outcomes, healthcare resources, and hospital budgets that could be achieved with even a modest (10-percentage point) increase in utilization of ready-to-use 3CBs in preterm neonates receiving PN across seven European countries.

## 2. Materials and Methods

### 2.1. Study Design

A cost-consequences analysis was performed to assess PN use in preterm babies born in Belgium, France, Germany, Italy, Portugal, Spain, and UK. In these countries, around 265,000 premature neonates (<37 weeks gestation) are estimated to be born annually [37,38,39,40,41,42,43,44], of whom approximately 133,000 (52%) receive PN [45]. A deterministic model was developed with inputs derived from an existing published budget-impact model [46]. The model estimated the national clinical outcomes, expressed as significant or severe harm, associated with compounding errors (i.e., any protocol deviation, such as wrong dose, wrong base solution, or ingredient omission that lead to temporary harm or permanent harm), and BSIs; national hospital labor time and costs associated with PN-related activities (prescribing, compounding, monitoring, stock management, requisition, and quality control); and total national hospital budget impact. The PN compounding methodologies assessed were hospital pharmacy manual, hospital pharmacy partly automated, ward, outsourced automated, industry provided non-3CBs, and ready-to-use 3CBs. Significant harm was defined as resulting in temporary harm requiring intervention and/or prolonged hospitalization. Severe harm was defined as resulting in permanent harm either requiring intervention to sustain life or causing death. Projections were based on increasing utilization of ready-to-use 3CBs by 10 percentage points and permutating other PN preparation methods accordingly, with the greatest changes assumed to derive from pharmacy manual and ward compounding (80% of share redistributed). Thus, we assumed that the greatest changes would be in pharmacy manual and ward compounding.

### 2.2. PN Use

Interviews in 12 hospitals in 2012 in Belgium, France, Germany, and the UK (three university hospitals, six general hospitals, two regional hospitals, and one private clinic) provided data on the most frequently prescribed formulations, average bottle or bag size, number of PN administration days, staff time required for compounding, and PN compounding method share, based on 49,922 pediatric PN bags administered [45,46]. Data on the annual preterm neonate population were directly extracted from governmental databases and official statistics reporting birth rates at different gestational ages or calculated by applying published prevalence rates of different preterm stages to overall national birth rates [37,38,39,40,41,42,43,44].

### 2.3. Compounding Errors and BSIs

For all countries, except the UK, the estimated baseline compounding error rates were set at 37% for pharmacy manual, 22% for pharmacy partly automated, 37% for ward, 1.7% for outsourced automated, and 1% for each of industry provided non-3CBs and ready-to-use 3CBs [21,22,23]. Input from industry experts and the literature indicated that strict local regulations in the UK equalized compounding errors across all compounding methodologies to 0.5% [47] except ward, for which the error rate was set at 37%. Across countries, rates of significant and severe harm associated with compounding errors were set at 3.5% and 0.4%, respectively [48].

BSI rates were set at 0.66% per PN-day for pharmacy manual compounding [49]. The rate was increased by 10 percentage points from this value for ward compounding and decreased by 10 percentage points for all pharmacy compounding and outsourced automated compounding, according to local industry healthcare experts’ opinions. For industry provided non-3CB and ready-to-use 3CB PN methodologies, BSI rates were decreased by 19%, based on the published literature [50].

### 2.4. Labor

Evidence in the literature suggests that ready-to-use 3CB solutions reduce the time spent by healthcare professionals on PN compounding [48,49]. To enable comparison, interviews with healthcare providers were used to derive the numbers of staff involved in prescribing, compounding (set-up time and performance), monitoring, stock management, requisition, quality control, training, and the time spent on these PN-related activities per role [44,45]. The data were validated by local and national industry experts. Annual salaries and compensation costs for hospital personnel were extracted from accessible official databases and literature sources [51,52,53,54,55,56,57]. Labor costs, time, and staff numbers were considered for senior pediatricians, senior pharmacists, pharmacist or medical-laboratory assistants, and specialized nurses.

### 2.5. Budget Impact

Budget impact was calculated based on clinical risks, costs of PN preparation (labor and supplies), and salaries for full time positions in all health-care categories.

BSI and compounding error costs were derived from Payne N.R. et al. [58] Karnon et al. [59] and the UK NHS National Schedule of Reference Costs 2016–2017 [60]. These were adjusted for inflation and purchasing power across the countries.

PN costs were calculated for the most frequently compounded all-in-one formulation (including carbohydrates, amino acids, lipid emulsions, electrolytes, vitamins, and trace elements) and adjusted per methodology. As it is possible for hospitals to negotiate reductions from list prices with manufacturers, where the net pricing remains confidential, we applied an assumed 25% reduction to list prices for PN ingredients, consumables, and ready-to-use 3CBs or used an average selling price. For other hospital compounding methods, the costs of ingredients per bag or bottle were calculated by multiplying the individual unit prices by the volume used per bag. The following bag sizes by birthweight were used, representing the most widely used range [42]: 150 mL for ≤1000 g; 220 mL for 1001–1500 g; 250 mL for 1501–2500 g; and 250 mL for >2500 g. With these ranges, the estimated average weighted hospital compounded nutrition costs, adjusted for inflation and adapted per local industry expert input, was €32 per bag/bottle. Costs associated with the discarding of partially used medication packages (e.g., due to hanging time restrictions) were disregarded.

Costs for consumables were obtained from wholesalers and laboratory distributor databases. Categories were disposable materials (including bags, bottles, needles, swabs, transfer systems, syringes, cannulas, catheters, and labels), garments (including sterile gloves, disposable headwear, masks and overshoes, clean room suit), and antiseptics.

For hospital labor costs, we identified salaries for senior pediatricians, senior pharmacists, pharmacist or medical-laboratory assistants, and specialized nurses per country. Salaries showed wide variability across countries, from €174,413 in the UK to €90,383 in Portugal for senior pediatricians; from €102,984 in Germany to €40,482 in Portugal for pharmacists; from €48,636 in the UK to €25,000 in Italy for nurses; and from €62,258 in Germany to €13,000 in Portugal for medical-laboratory assistants. Therefore, to estimate the change across all countries associated with a 10-percentage point increase in utilization of ready-to-use 3CBs, compounding time in minutes was multiplied by annual remuneration value per minute. The results were then divided by annual work time in minutes to estimate changes in the numbers of staff per role working full time on PN-related activities.

### 2.6. Sensitivity Analyses

In total, four one-way sensitivity analyses with highly conservative assumptions were conducted on the base case of a 10-percentage point change in utilization of ready-to-use 3CBs to test the uncertainty of selected variables and model the magnitude of impact of each on the total national hospital budget impact versus our baseline assumptions. Two analyses were done for in-hospital PN methodologies (pharmacy manual, pharmacy automated, and ward) assuming (1) an 80% lower compounding error rate and an 8% lower BSI error rate and (2) 50% lower nutrition ingredient/consumable costs. A third (3) analysis assumed overall 25% lower hospital labor costs and the last analysis (4) showed the effect of only 15% rebated ready-to-use 3CB acquisition costs.

## 3. Results

### 3.1. Compounding Method Share

The current PN compounding method share was estimated to be 43% for pharmacy manual, 16% for pharmacy partly automated, 22% for ward, 9% for outsourced, 3% for industry provided non-3CBs, and 7% for ready-to-use 3CBs (Table 1) based in literature and local industry experts input [45,46,61]. By increasing the ready-to-use 3CBs share by 10 percentage points and adjusting the distribution of other methods accordingly, with the greatest changes assumed to derive from pharmacy manual and ward compounding (80% of share redistributed), the estimated proposed PN compounding method share would change into 39% for pharmacy manual, 15% for pharmacy partly automated, 18% for ward, 9% for outsourced, 3% for industry provided non-3CBs, and 17% for ready-to-use 3CBs (Table 1).

### 3.2. PN-Associated Error Rates

Based on the estimated compounding error rates across all countries, with the current PN method share compounding errors leading to significant harm would be caused in >13,500 preterm neonates and leading to severe harm in >1500. BSIs were estimated to be seen in >10,400 preterm neonates (Table 2). After increasing utilization of ready-to-use 3CBs by 10 percentage points, these numbers would be reduced by 1575 for significant errors and 179 for severe errors (both 11.6%) and by 280 (2.7%) for BSIs (Table 2).

### 3.3. Labor Time

It was estimated that increasing ready-to-use 3CBs use by 10 percentage points would reduce labor time in all hospital roles assessed. Across all countries, the time saved would be equivalent to 41 specialized nurses (11.9% change), 29 (9.3%) senior pharmacists, 26 (9.9%) pharmacist assistants, and 22 (9.2%) senior pediatricians working full time (Table 3).

### 3.4. National Hospital Budget Impact

Based on the current PN compounding method share, the total hospital budget across all countries was estimated to be €260,329,814 per year. Increasing the use of ready-to-use 3CBs by 10 percentage points would increase proposed nutrition costs, but estimated cost-offsets due to avoided compounding errors, BSIs, and labor would reduce the total cross-country hospital budget impact by €8,960,601 (3.4%; Table 4).

### 3.5. Sensitivity Analyses

In the sensitivity analyses, the modeled effects of 80% lower compounding error rates and 8% lower BSI rates with in-hospital PN methodologies after a 10-percentage point increase in ready-to-use 3CBs, would reduce cost savings by 32% from € 8,960,601 to € 6,088,814 (Table 5). Similarly, if labor and hospital PN ingredient costs were reduced by 25%, savings would still be achieved, but would be 21% and 19% smaller, respectively (€7,093,589 and €7,293,793; Table 5). If the rebate for ready-to-use 3CBs were reduced to 15%, the total hospital budget impact saving would be 27% smaller (€6,577,485) compared with the modeled baseline scenario.

## 4. Discussion

This cost-consequences analysis demonstrated that even a modest 10-percentage point increase in the utilization of ready-to-use 3CBs for preterm neonates receiving PN could lead to substantial improvements in clinical outcomes and reductions in labor time and hospital budget impact. Importantly, rates of significant and severe harm due to compounding errors could decline notably by around 10–13% and BSIs by 2–3%. The sensitivity analyses, which included highly conservative assumptions, supported the findings, with benefits remaining substantial for all outputs.

For a complex medical process like PN compounding, in which multiple stakeholders are involved, it is essential not to limit comparative assessments to costs alone. In view of the fragility of the preterm population, a broader assessment needs to include clinical risks and other factors associated with different PN methodologies, such as time. Furthermore, the inclusion of clinical outcomes and labor is important to ensure a meaningful depiction of hospital processes, potential effectiveness gains, and costs involved. This study showed that the reduced labor associated with increased utilization of ready-to-use 3CBs freed up the equivalent of 119 full-time staff at multiple levels. The potential costs, time, and resources saved could be used to optimize hospital care in other areas that may lead to further hospital cost-savings and efficiencies gain. We chose to assess only a small change in practice because we felt that a 10-percentage point change to 3CBs would be widely achievable.

Human error has been identified as an important contributor to intravenous medication errors [14]. It was estimated here that a 10-percentage point increase in the utilization of ready-to-use 3CB PN could reduce the risk of temporary or permanent harm due to compounding errors by nearly 12% each. Risk of infection may be lowered directly by limiting the number of overall handling steps [25,26]. Finally, the time saved might have indirect benefits by allowing more time for checks and measures that could further reduce risks.

Several studies have demonstrated that a unique ready-to-use standardized PN not only may be easily used in all very preterm infants requiring PN but also can improve nutritional intakes and postnatal growth and provide good biological homeostasis [33,34,62,63,64]. One observational cohort study has also showed that the culture-proven BSI rate was significantly reduced by more than 50% when a 3CB was used in very premature infants [33]. In a systematic review, Mena and colleagues [65] found that ready-to-use 3CBs were associated with benefits in term and pre-term neonates, including reduced risks of infections and prescription errors, and ease of use, along with potentially reduced costs, while providing an adequate supply of nutrients and generating growth within the expected range. National and international guidelines [27,28,29,30] recommend that ready-to-use 3CBs be considered for first-line use. These recommendations and the findings of this study suggest that a much greater proportion of PN could be provided as ready-to-use 3CBs. Nevertheless, very high proportions of hospitals continue to order extemporaneously compounded PN and do not meet the guidelines for nutritional requirements [16,19].

### Limitations

This study has several limitations. Firstly, the estimates for errors associated with PN compounding are difficult to establish because of a wide variance in the reported literature. Therefore, the same error rates were applied in all studied countries except the UK, where values are notably different [47]. Other features we wished to assess were also poorly reported and, therefore, we consulted local industry experts to check that the data we found matched their experience. Additionally, there is a risk that BSI could be “double-counted”, as it is not always possible to distinguish whether they are direct consequences of compounding errors. Therefore, our findings might have overestimated or underestimated effects for individual countries because actual error rates are likely to vary widely, especially since compounding conditions in hospital pharmacies are likely not be the same as those 20 years ago. However, even with the very conservative assumptions in the sensitivity analysis, the effects of substitution with ready-to-use 3CBs on compounding errors and BSI rates remained substantial. To increase the accuracy of estimates, ethnographic research would be useful to identify the exact variations of error rates in each country and across hospitals.

Secondly, protocols for compounding are likely to differ between individual hospitals and between countries and, therefore, the labor variables might also vary substantially. To account for these potential differences, the impact of an increased use of ready-to-use 3CBs if hospital PN labor costs were 25% lower than in the base-case model was tested. Although the cost savings were reduced by 21%, still the time saved would be equivalent to nearly 89 staff working full time on compounding (i.e., *n* = 118 discounted by 25%).

Thirdly, individual hospitals and hospital groups are likely to have different procurement processes and negotiating powers with distributors and wholesalers. To consider the impact of this potential variance, we tested for 50% discounted onsite PN ingredients costs or limited discounts for ready-to-use 3CBs to 15%. The budget impacts were lessened by 19% and 27%, but the savings remained significant in most of the countries.

Fourthly, each hospital has its own unique PN environment that has likely changed from older publicly available assessments [45,46]. It was not possible to account for this variance in the model despite outreach to industry experts. While the estimated reductions in clinical outcomes, labor costs, and hospital budget impacts were aligned with findings in previous studies, they only represent national averages across countries. Large pan-European surveys, covering a substantial proportion of the hospital PN compounding universe are needed to assess current changes in PN compounding method share at more granular levels and take specific PN processes into account (e.g., involvement of health care staff at different seniority levels, utilization of PN methodologies, local protocols followed, etc.). For example, we assessed senior pharmacists because they are decision-makers, but in some hospitals, they will oversee junior pharmacist performing the compounding, spending considerably less time on this activity than senior pharmacists in other facilities. As noted in the study, we had to approach industry experts several times to provide or validate data, which could have led to bias on several levels. Obtaining standardized data from across countries through a dedicated evidence generation initiative, potentially including ethnographic components would mitigate this risk.

Finally, it was not possible to address all potential “hard” efficacy endpoints, such as correlation of different formulations with long-term growth rates, mental development, or even short-term mortality, due to limited data availability. We chose to concentrate on errors rates and BSI as indicators of efficacy due to the status of PN as a high-alert medication (i.e., has a heightened risk of causing significant harm to patients) [17]. Ultimately, well-designed prospective or retrospective evidence-generation initiatives are needed to assess robustly and holistically the comparative short-term and long-term clinical, resource, and economic implications of the main PN product methodologies available.

## 5. Conclusions

The gastrointestinal immaturity and clinical instability of premature neonates imply they may frequently require PN, especially very premature infants. There is wide variability in PN protocols and compounding methods across Europe, and many institutions do not fully align with international guidelines. In this international study, cost-consequences modeling indicates that exchanging even a small percentage of PN compounded in the hospital for ready-to-use 3CBs could provide substantial clinical benefits to preterm neonates and resource and cost savings to hospitals. The risks of overestimation and underestimation posed by the shortage of robust data were well accounted for in our sensitivity analyses, which confirmed the potential for substantial gains and improvements. However, given the highly dynamic and heterogenous PN hospital compounding landscape, additional evidence generation is required. In support of these goals, policies need to be developed to further improve PN protocols for preterm neonates. Implementation of programs that assess and implement methods to reduce morbidity and mortality among very and extremely premature neonates and the economic and resource burdens on hospitals should be prioritized at organization, health authority, and governmental levels [66].

## Figures and Tables

**Table 1:** Estimated current and proposed national PN Method Share Distribution.

	Current PN Compounding Method Share *								Proposed PN Compounding Method Share *							
	All	Belgium	France	Germany	Italy	Portugal	Spain	UK	All	Belgium	France	Germany	Italy	Portugal	Spain	UK
Pharmacy manual	43%	45%	24%	19%	48%	53%	72%	43%	39%	41%	22%	16%	45%	50%	64%	35%
Pharmacy partly automated	16%	10%	24%	19%	9%	24%	5%	21%	15%	8%	23%	18%	8%	22%	4%	20%
Ward	22%	14%	18%	58%	40%	23%	0%	0%	18%	10%	12%	52%	34%	18%	0%	0%
Outsourced automated	9%	0%	9%	0%	0%	0%	18%	36%	9%	0%	8%	0%	0%	0%	17%	35%
Industry provided non-3CBs	3%	0%	19%	4%	1%	0%	0%	0%	3%	0%	19%	4%	1%	0%	0%	0%
Ready-to-use 3CBs	7%	31%	7%	0%	3%	0%	5%	0%	17%	41%	17%	10%	13%	10%	15%	10%

* Due to rounding, some totals may not correspond. Abbreviations: 3CBs = three-chamber bags. PN = parenteral nutrition.

**Table 2:** Estimated national clinical implications of a 10-percentage point increase in ready-to-use 3CBs.

	Significant Compounding Errors *			Severe Compounding Errors ^†^			Bloodstream Infections		
	Current	Proposed	Impact	Current	Proposed	Impact	Current	Proposed	Impact
Belgium	719	622	−98	82	71	−11	532	515	−16
France	3554	3027	−527	406	346	−60	2958	2869	−89
Germany	4340	3899	−441	496	446	−50	2583	2513	−70
Italy	2085	1877	−208	238	215	−23	1169	1137	−32
Portugal	875	788	−87	100	90	−10	483	471	−12
Spain	1940	1726	−214	222	197	−25	1273	1244	−29
UK	43	43	0 ^‡^	5	5	0 ^‡^	1446	1415	−32
All	13,556	11,982	−1575	1549	1370	−179	10,444	10,164	−280
Percentage change			11.6%			11.6%			2.7%

* Defined as resulting in temporary harm requiring intervention and/or prolonged hospitalization. ^†^ Defined as resulting in permanent harm requiring intervention to sustain life or causing death. ^‡^ Due to strict regulations, the UK may represent a best-case example in terms of compounding error rates. Abbreviation: 3CBs = three-chamber bags.

**Table 3:** Estimated national effects of a 10 percentage point increased use of ready-to-use 3CBs on annual numbers of full-time equivalent * hospital staff spending time on compounding across different roles.

	Senior Pediatrician			Senior Pharmacists			Specialized Nurses			Pharmacist Assistants		
	Current	Proposed	Impact	Current	Proposed	Impact	Current	Proposed	Impact	Current	Proposed	Impact
Belgium	10.1	9.0	−1.1	16.8	15.0	−1.8	12.1	10.2	−1.9	14.3	12.6	−1.8
France	63.2	56.1	−7.1	80.2	74.9	−5.3	84.6	65.5	−19.1	64.1	59.7	−4.4
Germany	62.4	57.3	−5.1	49.9	44.7	−5.2	176.6	162.7	−13.9	39.9	35.5	−4.4
Italy	28.0	25.6	−2.4	36.2	33.7	−2.5	56.0	49.4	−6.6	30.9	28.9	−2.0
Portugal	12.3	11.2	−1.1	20.9	19.5	−1.4	14.1	11.8	−2.3	17.3	16.3	−1.0
Spain	30.2	27.7	−2.5	57.7	51.0	−6.7	1.0	3.0	2.0	50.2	44.4	−5.8
UK	37.8	34.8	−3.0	55.3	48.8	−6.5	0.0	0.7	0.7	45.8	38.8	−7.0
Full-time equivalent staff	244	222	−22	317	288	−29	344	303	−41	262	236	−26
Percentage change			9.0%			9.2%			11.9%			9.9%

* A full-time equivalent is defined as the ratio of the total number of paid hours per year performing compounding to the total number of annual working hours for an employee (i.e., one full-time equivalent is equivalent to one employee working full-time on compounding). Abbreviations: 3CBs = three-chamber bags.

**Table 4:** Estimated total national hospital budget impact with a 10-percentage point increase in utilization of ready-to-use 3CBs.

	Total National Hospital Budget Impact		
	Current	Proposed	Impact
Belgium			
Compounding errors *	€1,395,592	€1,206,029	−€189,563
BSI	€5,058,364	€4,901,890	−€156,473
Nutrition ^†^	€2,532,135	€2,891,959	€359,824
Labor	€3,999,825	€3,536,499	−€463,326
Total	€12,985,915	€12,536,378	−€449,538
France			
Compounding errors *	€6,306,157	€5,371,408	−€934,749
BSI	€25,728,804	€24,955,286	−€773,517
Nutrition ^†^	€20,852,047	€21,924,863	€1,072,816
Labor	€18,798,036	€16,706,289	−€2,091,747
Total	€71,685,043	€68,957,846	−€2,727,197
Germany			
Compounding errors *	€7,618,181	€6,843,597	−€774,585
BSI	€22,227,948	€21,630,643	−€597,306
Nutrition ^†^	€19,851,747	€20,293,467	€441,720
Labor	€23,911,659	€21,782,293	−€2,129,366
Total	€73,609,535	€70,549,999	−€3,059,536
Italy			
Compounding errors *	€3,222,699	€2,901,138	−€321,561
BSI	€8,860,322	€8,616,838	−€243,484
Nutrition ^†^	€8,607,327	€8,876,975	€269,648
Labor	€10,238,265	€9,400,403	−€837,862
Total	€30,928,613	€29,795,354	−€1,133,259
Portugal			
Compounding errors *	€767,989	€692,011	−€75,978
BSI	€2,077,154	€2,025,068	−€52,086
Nutrition ^†^	€1,608,427	€1,930,824	€322,397
Labor	€2,635,936	€2,398,603	−€237,334
Total	€7,089,506	€7,046,505	−€43,001
Spain			
Compounding errors *	€3,330,748	€2,963,104	−€367,644
BSI	€10,714,234	€10,458,804	−€255,430
Nutrition ^†^	€6,726,140	€7,304,860	€578,720
Labor	€6,312’992	€5,739,594	−€573,398
Total	€27,084,114	€26,466,361	−€617,752
UK			
Compounding errors *	€73,466	€73,466	€0
BSI	€12,058,976	€11,792,978	−€265,997
Nutrition ^†^	€12,748,486	€13,219,532	€471,046
Labor	€12,066,158	€10,931,142	−€1,135,016
Total	€36,947,087	€36,016,769	−€930,318
All	€260,329,814	€251,369,212	−€8,960,601
Percentage change			−3.4%

* Compounding error costs are those associated with any protocol deviation. ^†^ Nutrition costs that are associated with parenteral nutrition and its preparation overall (including acquisition costs). Abbreviations: BSI = bloodstream infection; 3CBs = three-chamber bags.

**Table 5:** Sensitivity analyses.

	Base-Case Model *	Sensitivity Analysis 1 (Compounding Errors and BSI) ^†^			Sensitivity Analysis 2 (Labor Costs) ^‡^			Sensitivity Analysis 3 (Nutrition Costs) ^§^			Sensitivity Analysis 4 (Ready-to-use 3CBs 15% Rebate) ^#^		
	THBI	Current	Proposed	Impact	Current	Proposed	Impact	Current	Proposed	Impact	Current	Proposed	Impact
Belgium	−€449,538	€11,522,241	€11,286,605	−€235,635	€11,985,959	€11,652,253	−€333,706	€12,635,433	€12,225,168	−€410,265	€13,183,340	€12,797,488	−€385,852
France	−€2,727,197	€65,784,781	€64,018,429	−€1,766,352	€66,985,535	€64,781,274	−€2,204,260	€66,760,241	€64,493,586	−€2,266,656	€72,243,081	€70,313,080	−€1,930,001
Germany	−€3,059,536	€65,703,811	€63,459,557	−€2,244,254	€67,631,620	€65,104,426	−€2,527,195	€67,185,029	€64,621,532	−€2,563,497	€73,609,535	€70,666,729	−€2,942,806
Italy	−€1,133,259	€27,726,369	€26,924,294	−€802,076	€28,369,047	€27,445,253	−€923,794	€28,155,007	€27,233,740	−€921,266	€31,250,332	€31,266,066	€15,735
Portugal	−€43,001	€6,345,313	€6,379,349	€34,036	€6,403,522	€6,446,855	−€16,333	€6,553,364	€6,553,353	−€11	€7,089,506	€7,106,687	€17,181
Spain	−€617,752	€23,739,659	€23,513,116	−€226,543	€25,505,866	€25,031,463	−€474,403	€25,238,396	€24,774,218	−€464,178	€27,155,752	€26,681,278	−€474,475
UK	−€930,318	€36,504,574	€35,565,584	−€847,990	€33,930,547	€33,283,983	−€646,564	€34,468,968	€33,801,048	−€667,920	€36,947,087	€36,069,820	−€877,267
All	−€8,960,601	€237,326,748	€231,237,934	−€6,088,814	€240,839,096	€233,745,507	−€7,093,589	€240,996,438	€233,702,645	−€7,293,793	€261,478,633	€254,901,148	−€6,577,485
% of baseline	100%			68%			79%			81%			73%

* Use of ready-to-use 3CBs increased by 10 percentage points and use of other methods were adjusted accordingly. ^†^ Compounding error rates for onsite compounding (pharmacy manual, pharmacy automated, and ward) were reduced by 80% and BSI rates were reduced by 8%. ^‡^ Hospital staff wages were reduced by 25% (or taken to be equivalent to 25% less time spent for PN compounding at baseline wages). ^§^ Nutrition costs for onsite hospital compounding were reduced to 50% from baseline. ^#^ Ready-to-use 3CBs only rebated by 15%. Abbreviations: BSI = bloodstream infection; 3CBs = three-chamber bags; THBI = total hospital budget impact.
